# Multiple endo bronchial lipoma: a rare case report

**DOI:** 10.1186/s12890-020-01287-4

**Published:** 2020-09-22

**Authors:** Shunjin Zhao, Yuexiang Shui, Zhong Dai

**Affiliations:** Department of Respiratory Medicine, Lanxi People’s Hospital, No. 1359, Xishan Road, Lanxi, Jinhua, 321100 Zhejiang Province China

**Keywords:** Bronchoscopy, Case report, Lipoma, Endobronchial lipoma

## Abstract

**Background:**

Endobronchial lipoma is an extremely rare benign tumor, which is generally located in the first three subdivisions of the tracheobronchial tree. According to the existing literature, all endobronchial lipomas are single (one per patient). Here, we report a rare case in which the patient presented with two endobronchial lipomas in the same patient, and underwent a bronchoscopic tumor resection in the left main bronchus and the left lower bronchus. Both tumors were pathologically confirmed as endobronchial lipoma.

**Case presentation:**

A 52-year-old Chinese man presented at the clinic reporting a mild cough with yellow color sputum and exertional dyspnea for 2 weeks. He was a heavy smoker (45 pack-years). Chest auscultation demonstrated faint wheezing in left lower lobe. Computed tomography (CT) revealed two low-density endobronchial masses located in the middle segment of the left main bronchus and the posterior basilar segmental bronchus of the left lower lobe. The neoplasms measured a CT-attenuation value of -70HU, −98HU in density with air trapping and atelectasis in the segmental bronchus of the left lower lobe. The patient underwent interventional bronchoscopic management to remove the neoplasms by using an electrosurgical snare, cryotherapy, and electrocautery. The locations of the neoplasms were confirmed at the left main bronchus and the superior segment of the left lower lobe during bronchoscopic intervention. Histopathological examination confirmed that both tissues were consistent with lipomas. After 18 months of follow-up, the patient was free of symptoms and CT revealed that bronchiectasia remained in the superior segment of the left lower lobe; however, no mass lesion was present in the left bronchus.

**Conclusions:**

This case suggests that an endobronchial lipoma can present as multiple lesions, and both proximal and distal types can simultaneously occur in the same patient. Thus, these findings help us further understand the biology of endobronchial lipomas.

## Background

Endobronchial lipoma is an extremely rare benign tumor, comprising approximately 0.1–0.5% of all bronchial tumors [1]. In most cases, tumors are located in the first three subdivisions of the tracheobronchial tree [2]. When they become large enough, these tumors can lead to endobronchial obstruction, thereby causing atelectasis and recurrent pneumonia, even irreversible lung damage if undiagnosed early. Endobronchial lipomas are more common in men and in the right lung [3]. Histologically, endobronchial lipomas have been shown to contain numerous uniform adipocytes [4]. According to the existing literature, all endobronchial lipomas are single (one per patient). Here, we report a rare case in which a patient presented with two lesions, and underwent bronchoscopic tumor resection in the left main bronchus and the left lower bronchus. Both tumors were pathologically confirmed as endobronchial lipoma.

## Case presentation

**Fig. 1:**
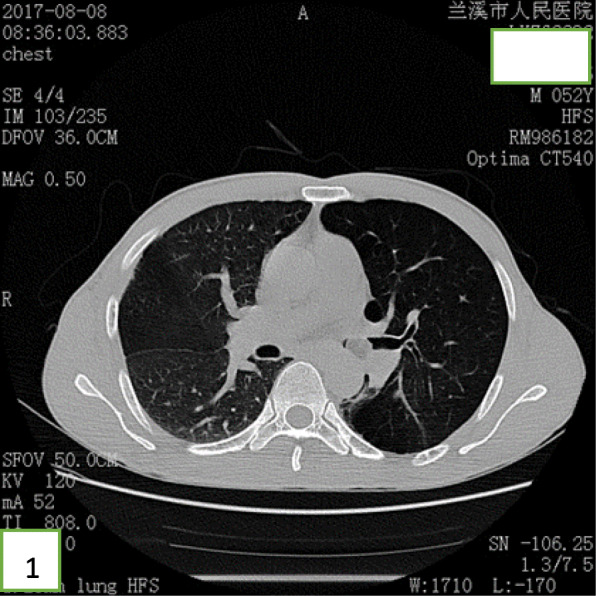
CT showing a low-density mass obstructing the left main bronchus, the CT value is -70HU

**Fig. 2:**
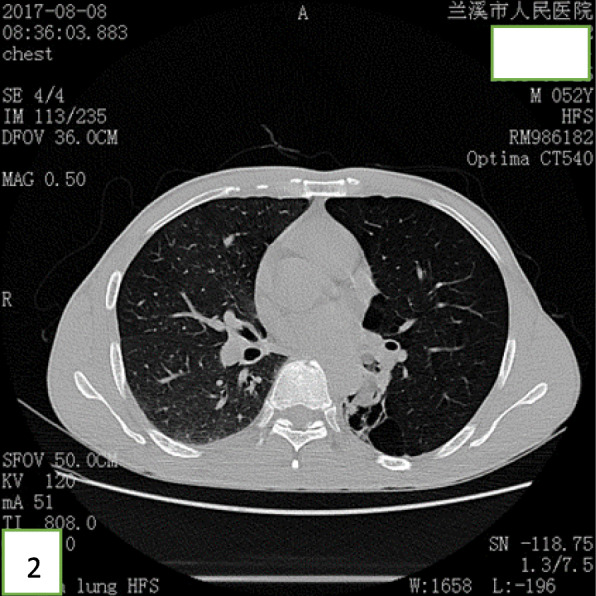
CT suggesting low-density mass obstructing the left lower lobe, the CT value is -98HU

**Fig. 3:**
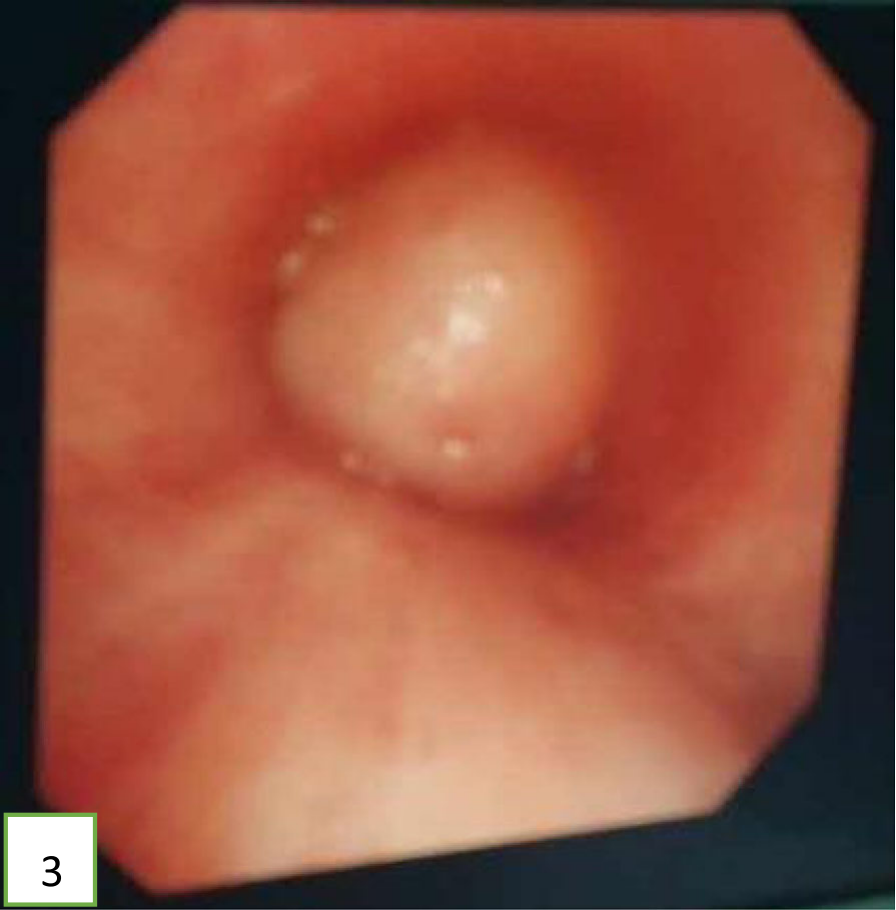
A spherical neoplasm growing in the left main bronchus with a smooth, soft, and red appearance

A 52-year-old Chinese man presented at the clinic reporting a mild cough with yellow color sputum and exertional dyspnea for 2 weeks. He denied any fever, chest pain, night sweats, or weight loss. He was a heavy smoker (45 pack-years) and had a history of a splenectomy for abdominal injury 23 years ago. After a failed 3 day-course of antibiotic treatment at the clinic, he was referred to our hospital for further examination and treatment. Upon physical examination, the body mass index (BMI) was 17.97, SpO_2_ was 98% when breathing ambient air, the respiratory rate was 20/min, and the blood pressure was 119/77 mmHg. Chest auscultation demonstrated faint wheezing in left lower lobe. A computed tomography (CT) scan was performed, which revealed two low-density endobronchial masses located in the middle segment of the left main bronchus and the posterior basilar segmental bronchus of the left lower lobe. The neoplasms measured the CT-attenuation value -70HU, −98HU (Figs. 1 and 2) in density with air trapping and atelectasis in the segmental bronchus of the left lower lobe. The complete blood count showed that the white blood cell count increased to 16 * 10^9/L, the platelet count increased to 549 * 10^9/L, whereas other blood cell counts were within the normal range. No abnormalities were observed in liver function, renal function, blood lipids, blood glucose, C-reactive protein, and erythrocyte sedimentation rate. The tumor markers alpha fetoprotein (AFP), carcinoembryonic antigen (CEA), carbohydrate antigen 125 (CA125) and carbohydrate antigen 19–9 (CA19–9) were in the normal range, and no acid-fast bacilli were found in sputum smears. Subsequently, the patient underwent a flexible bronchoscopy, which confirmed an exophytic spherical lesion that caused almost complete occlusion of the middle of the left main bronchus (Fig. 3), however, the cytological diagnosis failed by using bronchoscopic brushing cells.

**Fig. 4:**
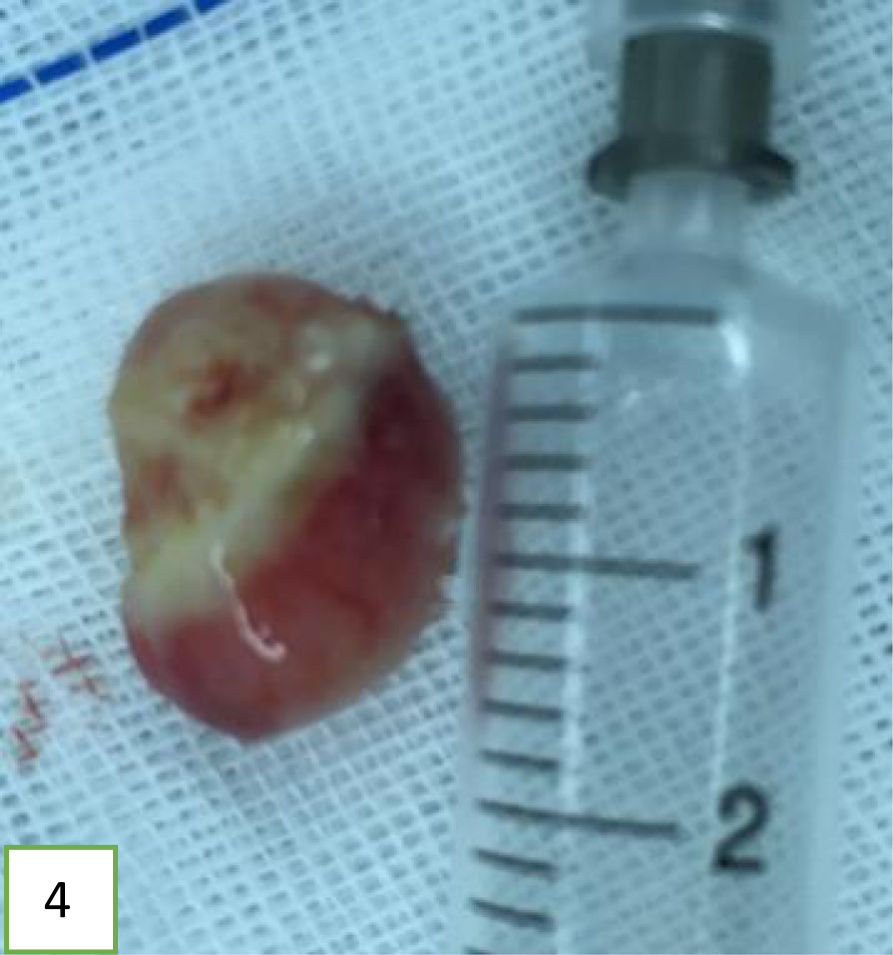
Neoplasm located in left main bronchus, about 2.0 × 1.3 × 0.9 cm in size, with an oval polypoid shape

**Fig. 5:**
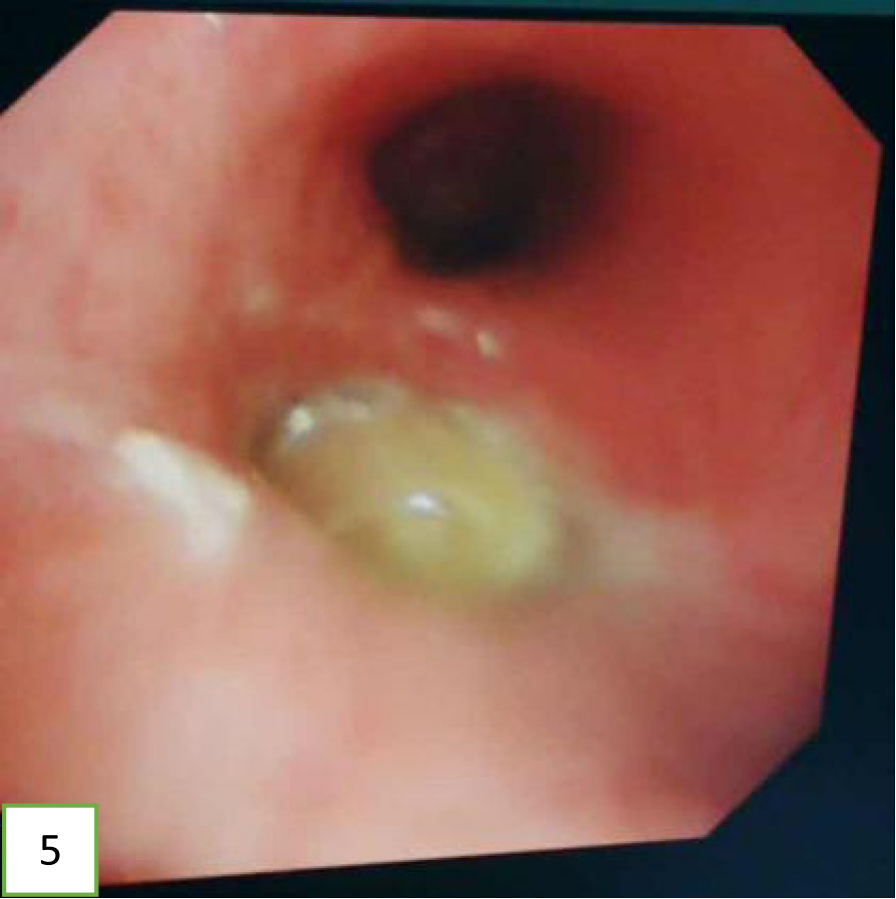
The left lower lobe bronchus is blocked by a neoplasm. Its surface is smooth, soft, has a light-yellow appearance, and is dumbbell shaped

**Fig. 6:**
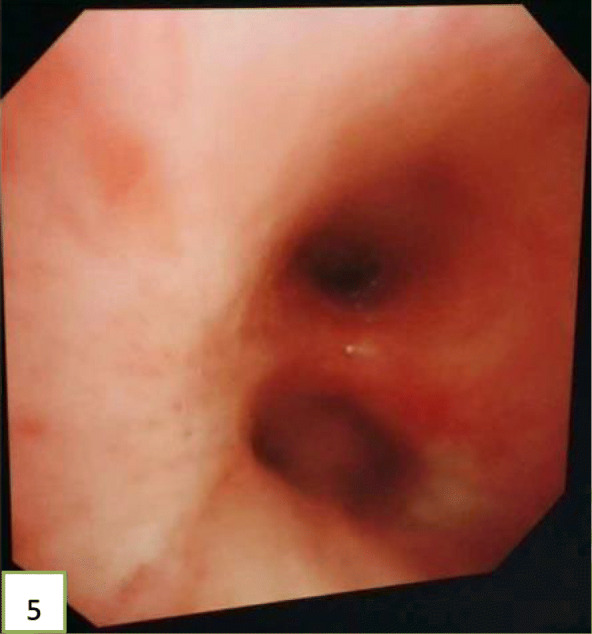
Image of left main bronchial tumor after resection

**Fig. 7:**
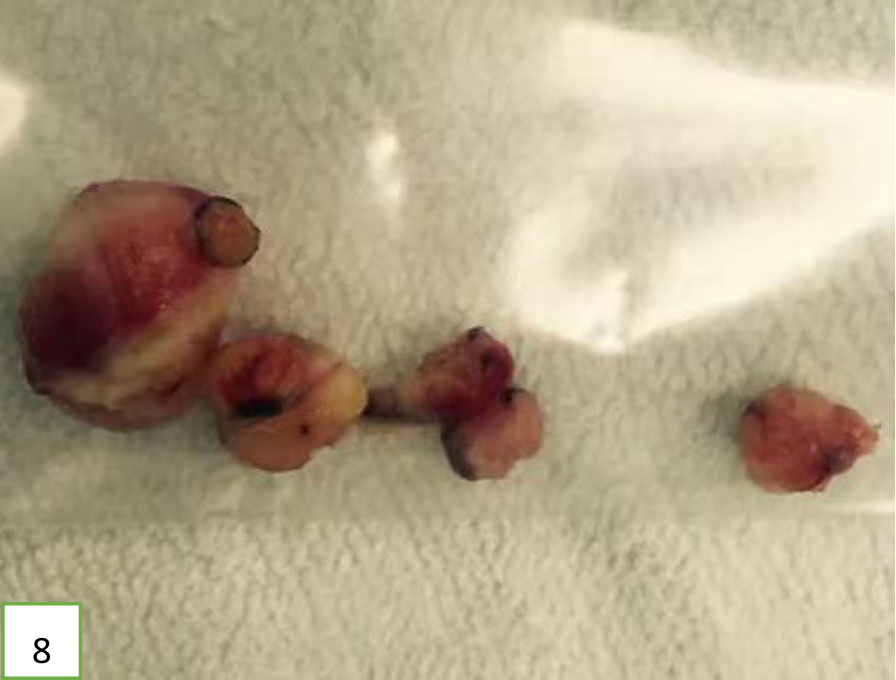
Five pieces of neoplasia tissues that have been removed from the left main bronchus and the lower left bronchus

**Fig. 8:**
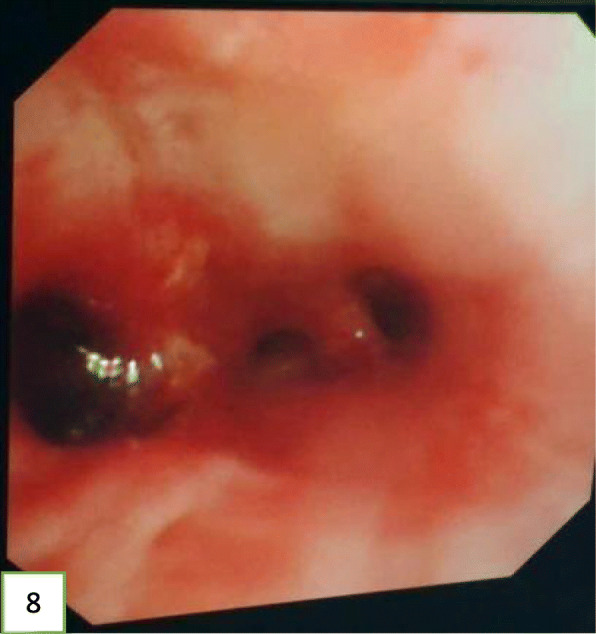
Image of the left lower lobe bronchial after tumor resection

**Fig. 9:**
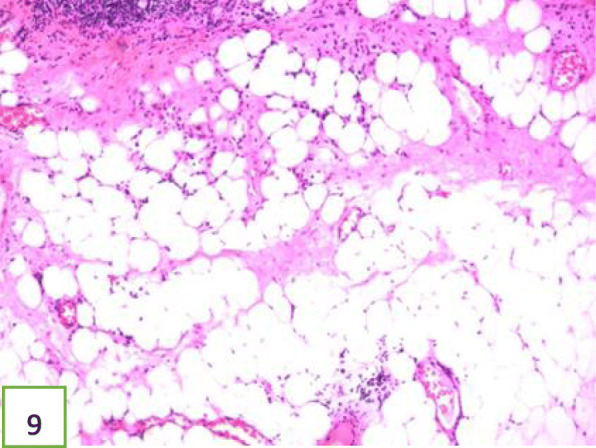
Histology of the neoplasm at low magnification demonstrating the pseudo-stratified ciliated columnar epithelium overlying the mature adipocytes and little fibrous tissue. The neoplasm in the left main bronchus is consistent with lipoma. Hematoxylin and eosin (HE) staining

**Fig. 10:**
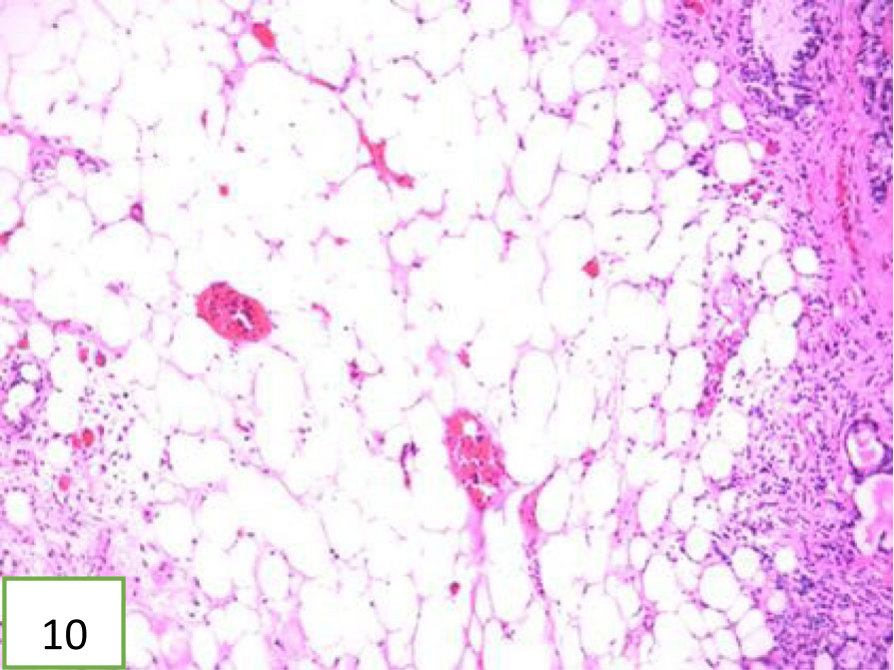
Histology of the neoplasm at low magnification demonstrating the pseudo-stratified ciliated columnar epithelium overlying mature adipocytes. The neoplasm in the left lower lobe is consistent with lipoma. HE staining

In view of the above, interventional bronchoscopic management was undertaken to remove the neoplasms. First, we therapeutically resected the neoplasm, of which the base was located in the lateral wall of the left main bronchus using electrosurgical snare under flexible bronchoscope (Fig. 3). Then, the spherical and pink neoplasms were taken out by cryotherapy (Fig. 4). Subsequently, another yellowish dumb-bell neoplasm was found in the superior segment of the left lower lobe (Fig. 5) and the main part of the neoplasm was removed (Figs. 6 and 7) by electrosurgical snare, electrocautery, and cryotherapy. Only the C branch of the superior segment of the left lower lobe could not be completely resected or ablated (Fig. 8) because the neoplasm originated distally. Histopathological examination confirmed that both tissues from the left main bronchus and the superior segment of the left lower lobe were consistent with lipoma (Figs. 9 and 10).

**Fig. 11:**
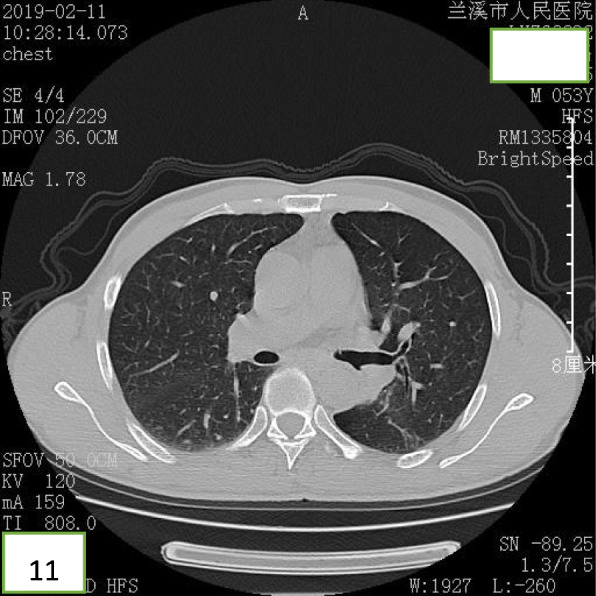
CT revealing that the left main bronchus and the left lower lobe were unobstructed, and that the left lower lobe showed bronchiectasis

**Fig. 12:**
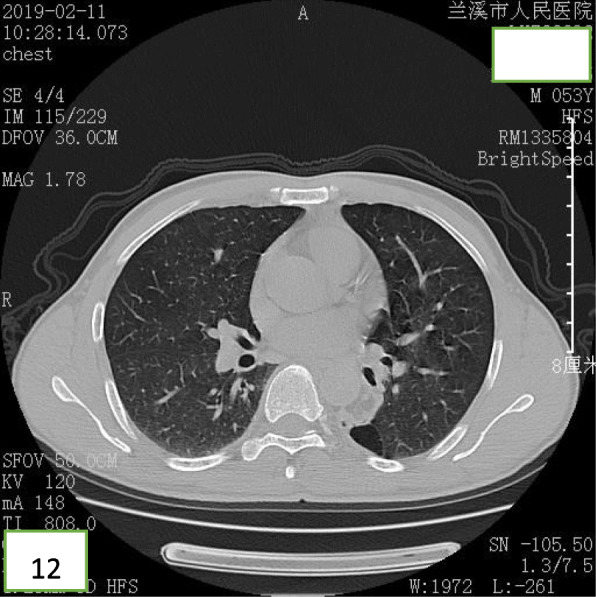
CT revealing that the left main bronchus and the left lower lobe were unobstructed, and that the left lower lobe showed bronchiectasis

After bronchoscopic intervention, the patient recovered and was discharged. After 18 months of follow-up, the patient was free of symptoms and CT revealed that bronchiectasia remained in the superior segment of the left lower lobe (Figs. 11 and 12), however, no mass lesion was present in the left main bronchus.

## Discussion and conclusion

Endobronchial lipoma is a type of rare benign tumor, which originates from fat cells located in peribronchial and submucosal tissues of the bronchi [5]. Although lipomas in connective tissue, such as subcutaneous tissue, can present as multiple lesions, lipomas in the bronchi are almost always present as a single lesion. The authors used ‘lipoma’ and ‘bronchus’ as keywords to search the PubMed Database, thereby retrieving all endobronchial lipomas with a clear pathological diagnosis that presented as a single lesion. The case reported here was unique in that two endobronchial lipomas were present in the same patient. According to the location, endobronchial lipomas are divided into two types [3]: 1. Proximal type: often occurs in the large bronchi, and is rich in normal adipose tissue, including trachea, carina, main bronchus, and bronchus intermedius [6]. 2. Distal type: located below the segmental bronchus, and originates from the surrounding bronchial wall adipose tissue, including segmental and subsegmental [7]. The case presented here was unique in that two endobronchial lipomas were present in one patient, and were located in the left main bronchus, and the superior segment of the left lower lobe, respectively. These findings suggested that an endobronchial lipoma can also present as multiple lesions, and both proximal and distal types can simultaneously occur in the same patient. It is necessary to compare the characteristics of this endobronchial lipoma case with previous lipomas because of its uniqueness.

For most cases, lipomatous neoplasms arise in the setting of lipomatosis and rarely involve the tracheobronchial tree [1, 5]. Endobronchial lipomas arise from submucosal fat in airways that contain cartilage and bronchial glands, and are usually covered by normal respiratory epithelium [8]. Most patients are men (80%) with a mean age of 60 years, and a significant smoking history [2]. These benign tumors typically occur in the large bronchi (first 3 subdivisions), and may lead to obstructive complications [9]. The patient suffering from endobronchial lipomas was a 52-year-old man who was a heavy smoker, which was consistent with the risk factors of lipomas for age, sex, and smoking history [9]. However, the patient’s BMI was only 17.96, which was not consistent with previous findings that obesity was a risk factor for endobronchial lipomas [8].

Endobronchial lipomas grow slowly and have different clinical manifestations [10]. The clinical symptoms are related to the location of the tumor and the severity of endoluminal obstruction [11]. Symptoms can remain clinically silent for a prolonged period when the tumor is small. As the tumor grows and significantly obstructs the lumen, it can lead to cough, sputum, fever, dyspnea, and other symptoms [11]. Nassiri et al. reported that atelectasis was present in 30% of cases, and that the majority of patients was symptomatic (63.2%) [12]. However, the patient in this case did not have clinical symptoms in the early stage until he developed post-obstructive pneumonia. Patients are often misdiagnosed as having asthma or chronic bronchitis and endobronchial lipomas can remain undetected for months or years [13]. Thus, to shorten the diagnostic time of endobronchial lipomas, CT and bronchoscopy should be performed early when a patient is actively undergoing unexplained cough, dyspnea, fever, or other symptoms.

At present, a chest CT and bronchoscopy are main approaches to diagnose endobronchial lipoma as the sensitivity of traditional X-ray examination is lower (66%) [14, 15]. The diagnosis can be suggested with fat attenuation on a chest CT and the lack of enhancement after contrast administration [16, 17], because the density of the lipoma is similar to that of normal adipose tissue, and the CT-attenuation value is between − 120 Hu and − 40 Hu. It is helpful to distinguish lipoma from other tumors by accurately measuring the CT-attenuation value of the tumor. In our case, we measured the CT-attenuation value of the neoplasms, which was within the CT-attenuation value range of the lipoma, and helped us make the diagnosis of endobronchial lipoma. In addition, a chest CT can accurately display the shape, size, location, degree of lumen stenosis, the relationship with the bronchial wall, and indirect obstructive signs of a tracheobronchial lipoma [8]. However, it is a challenge to distinguish endobronchial lipoma from bronchial hamartoma by CT, because a bronchial hamartoma is predominantly composed of adipose tissue. Therefore, a bronchoscopy is another important tool for the diagnosis of endobronchial lipoma, can visually show the characteristics of lipoma and biopsy, and can accurately determine the tumor’s location, shape, and degree of blockage of the tumors. In previous reports, it has been suggested that the diagnostic value of a bronchoscopy is very low because of the solid cystic tissue covering the surface of the bronchial lipoma [15]. Only one third of patients can be diagnosed by bronchoscopic biopsy [2]. However, in some studies it was reported that using a cryoprobe, large pieces of the tumor can be extracted, which can help overcome the limitation of a low diagnostic yield [18]. In addition, surgical excision can be performed to obtain pathological diagnosis when neither CT nor bronchoscopy is confirmed. In this case, the chest CT had accurately judged the quantity, size and CT-attenuation value of the tumors, but made an error in judging the location (CT indicated one of the lesions was in the posterior basilar segment of left lower lobe, however the bronchoscopic resection confirmed that the location was in the superior segment of the left lower lobe). These findings display that CT is a reliable method for the diagnosis of endobronchial lipoma even if there are minor faulty judgements. In this case, we failed in diagnosing the tumors by bronchoscopic brushing cytology, however, bronchoscopy was very important to differentiating between benign and malignant tumors since the shape, activity, surface condition, and obstruction degree of the tumors could clearly be observed under the bronchoscope, which provided a basis for the formulation of treatment.

Endoscopic features of tracheobronchial lipomas have been classified as follows. These tumors always appear with a smooth surface and are oval-shaped, only a few could be lobulated, and they are poorly vascularized with a yellow to rose appearance. Furthermore, the mobility of the tumors is in general mobile, it is rarely to see fixed one. Finally, the proportions of proximal and distal types are similar when it comes to the location of the tumors [8, 12]. The two endobronchial lipomas in our case were different in shape, color, and mobility, thereby suggesting that the characteristics of multiple endobronchial lipomas can be diverse, and more attention should be paid on it to avoid misdiagnosis.

The treatment of endobronchial lipomas includes two methods: bronchoscopic resection and surgery [19]. With the development of bronchoscopic interventional technology, bronchoscopic interventional therapy for benign central airway stenosis has been widely used [2, 4, 12, 15]. When compared with surgery, bronchoscopic resection can completely relieve the symptoms of patients, has a low risk, fewer complications, better patient tolerance, and would preserve lung tissue and lung function [2, 20]. Most cases of proximal endobronchial lipoma can be removed by bronchoscopy. There is variability in strategy, including both rigid/flexible bronchoscopy, and use of electrocautery, cryotherapy, argon plasma coagulation, laser, and/or mechanical debulking [4]. A rigid bronchoscope is preferred over a flexible bronchoscope because of the wider internal diameter and the higher airway safety [14]. Surgery is another method of treatment, and the most effective method, however, surgery has a greater risk for more complications, has a higher cost, and requires a patient to be fit enough to undergo surgical resection. However, surgical procedures should be reserved for patients with a possible coexistent malignant tumor, severe irreversible damage of the distal bronchus and lung tissue, distal type tumors growing around the bronchus, or technical difficulties during bronchoscopic procedure, such as multidirectional tumor growth [19, 21]. In this case, we removed both the proximal type and distal type lipoma using a flexible bronchoscope without any complications. Surgery had not been performed for the rest of tumor in the left lower lobe, because it did not cause serious damage in the distal bronchus and the lung. It was a slowly growing tumor without excess risk of malignant potential [2]. During a prolonged follow-up (18 months), no recurrence and new obstruction had occurred, and no aggravation of distal bronchiectasis and lung damage was observed. This demonstrated that endobronchial resection was effective and safe and preserved lung tissue in this subgroup of benign pulmonary tumors [12].

In summary, in this case we reported multiple endobronchial lipomas that coexisted proximal and distal, indicating a novel discovery of endobronchial lipoma, as all cases previously reported were single. We demonstrated that the clinical features, radiological features, endoscopic features, and histopathological features supported previous observations. Taken together, this case helps us further understand the biology of endobronchial lipomas.

## Data Availability

The data are available from the corresponding author on reasonable request.
